# The association between abdominal obesity and femoral neck bone mineral density in older adults

**DOI:** 10.1186/s13018-023-03654-3

**Published:** 2023-03-06

**Authors:** Jun Chen, Liming Zhu, Xiaocong Yao, Zhongxin Zhu

**Affiliations:** 1 Department of Endocrinology, Xiaoshan Affiliated Hospital of Wenzhou Medical University, Hangzhou, 311200 Zhejiang China; 2 Department of Osteoporosis Care and Control, Xiaoshan Affiliated Hospital of Wenzhou Medical University, Hangzhou, 311200 Zhejiang China; 3 Department of Clinical Research Center, Xiaoshan Affiliated Hospital of Wenzhou Medical University, Hangzhou, 311200 Zhejiang China

**Keywords:** Abdominal obesity, Waist circumference, Bone mineral density, Older adults, NHANES

## Abstract

**Background:**

The relationship between obesity and osteoporosis is complex, with contradictory findings reported. Our aim was to evaluate the association between waist circumference (WC), as an easy-to-determine clinical index of abdominal obesity, and femoral neck bone mineral density (BMD) among older adults, using the National Health and Nutrition Examination Survey (NHANES) database.

**Methods:**

Data of five NHANES cycles (2005–2010, 2013–2014, and 2017–2018), including 5801 adults aged ≥ 60 years, were used in the analysis. Weighted multiple regression analyses were performed to evaluate the association between WC and femoral neck BMD. Weighted generalized additive models and smooth curve fitting were further performed to characterize nonlinearities in the association.

**Results:**

There was a positive association between WC and femoral neck BMD in non-adjusted models. After adjusting for body mass index (BMI), the association became negative. On subgroup analysis stratified by sex, this negative association only existed for men. An inverted U-shaped curve relationship between WC and femoral neck BMD was further identified, with an inflection point at a WC of 95 cm for both men and women.

**Conclusions:**

Abdominal obesity is a negative predictor of bone health among older adults, independent of BMI. The association between WC and femoral neck BMD followed an inverted U-shaped curve.

## Introduction

Osteoporosis has become a serious problem globally, with its prevalence having increased to 21.7% among elderly individuals [1]. The rate of obesity is also increasing among aging adults [2], with 57.8% of older individuals projected to be overweight or obese by 2030 [3]. Although osteoporosis and obesity may occur together, the relationship between the two is complex, with contradictory views presented.

Obesity is generally classified as abdominal obesity, indexed by waist circumference (WC), and general obesity, quantified by the body mass index (BMI) [4]. A positive relationship between BMI and bone mineral density (BMD) has been reported, indicating that higher body weight may have a protective effect against osteoporosis [5]. Yet, a positive association between general obesity and bone fractures was revealed by recent literature [6]. Therefore, the effect of obesity on bone health is complicated and controversial, creating an “obesity paradox” [7]. Moreover, abdominal obesity is increasingly recognized as a key contributor to adverse health risks [8]. Yet, the association between abdominal obesity and BMD among older adults remains to be clarified. Moreover, as the proportion of elderly in the general population has increased, the incidence of hip fractures has risen [9], which usually requires surgical treatment and has been a leading cause of hospitalization in elderly patients [10, 11]. Accordingly, we aimed to evaluate the association between WC and femoral neck BMD among older adults, using the National Health and Nutrition Examination Survey (NHANES) database.

## Methods

### Study population

The NHANES survey uses a complex, multistage, probability sampling design to evaluate the health and nutritional status of the non-institutionalized population of the United States. The data of the NHANES survey are released in 2-year cycles. As the NHANES 2011–2012 and 2015–2016 cycles did not include femoral neck BMD data, we combined data from the following five cycles for analysis in our study: 2005–2010, 2013–2014, and 2017–2018. Among the 9788 adults, aged ≥ 60 years, identified in the database, we excluded those with missing WC or BMI data (n = 1291), missing femoral neck BMD data (n = 1219), and with a cancer history (n = 1477). After screening, the data from 5801 participants were included in the final analysis.

### Ethics statement

All NHANES protocols were approved by the National Center for Health Statistics (NCHS) Research Ethics Review Board and written informed consent was obtained from the participants for data collection and publication for research.

### Study variables

The exposure variable was WC, which was collected by trained health technicians in the Mobile Examination Center. WC was measured at the uppermost lateral border of the right ilium, to the nearest 0.1 cm. The outcome variable was femoral neck BMD, which was obtained by dual-energy X-ray absorptiometry (DXA; Hologic Inc., Bedford, MA USA) [12].

Additionally, the following data were collected as covariates in the analysis: age, sex, race, education level, income-poverty ratio, moderate recreational activities, alcohol use, smoking cigarettes, BMI, blood urea nitrogen, total protein, serum uric acid, serum glucose, serum phosphorus, serum calcium, and serum 25(OH)D. Details of the acquisition process of WC, BMD, and other covariates are available on the NHANES website (www.cdc.gov/nchs/nhanes/).

### Statistical analysis

As recommended by the analytical guideline edited by NCHS, we used appropriate sampling weights to ensure national representation. Weighted multiple regression analyses were used to evaluate the association between WC and femoral neck BMD. According to the STrengthening the Reporting of OBservational studies in Epidemiology (STROBE) guidelines[13], we employed four models: model 1, no adjustment; model 2, adjusted for age, sex, and race; model 3, model 2 plus adjustment of BMI; and model 4, adjusted for all covariates. To characterize nonlinearity in the association between WC and femoral neck BMD, weighted generalized additive models and smooth curve fittings were further performed. All analyses were performed using EmpowerStats software and R version 3.4.3, with a *P* < 0.05 considered statistically significant.

## Results

The weighted relevant characteristics of the 5,801 participants included in the analysis are presented in Table 1. Compared to women, men had higher levels of serum uric acid, glucose, blood urea nitrogen, total protein, WC, femoral neck BMD, and lower levels of serum phosphorus and calcium.

**Table 1:** The weighted characteristics of the subjects

	Men (n = 2,971)	Women (n = 2830)	*P* value
Age (years)	68.4 ± 6.5	69.4 ± 6.9	< 0.001
*Race (%)*			0.156
Non-Hispanic White	75.3	76.0	
Non-Hispanic Black	8.6	9.8	
Mexican American	5.3	4.7	
Other race	10.8	9.5	
*Education level (%)*			< 0.001
Less than high school	20.7	20.4	
High school	24.8	29.3	
More than high school	54.5	50.2	
Income-poverty ratio	3.2 ± 1.6	2.9 ± 1.6	< 0.001
Body mass index (kg/m^2^)	28.6 ± 4.8	28.3 ± 5.8	0.038
*Moderate recreational activities (%)*			0.105
Yes	36.5	34.6	
No	46.0	48.8	
Not recorded	17.6	16.7	
*Average drinks/day during past 12 months (%)*			< 0.001
1 drink	27.6	36.2	
2–4 drinks	32.3	17.4	
More than 4 drinks	4.9	0.8	
Not recorded	35.3	45.6	
*Average cigarette/day during past 30 days (%)*			< 0.001
1–3 cigarettes	1.4	1.7	
4–10 cigarettes	3.4	3.1	
More than 10 cigarettes	8.7	5.3	
Not recorded	86.5	89.9	
Blood urea nitrogen (mmol/L)	6.0 ± 2.3	5.7 ± 2.2	< 0.001
Total protein (g/L)	70.3 ± 4.6	69.9 ± 4.6	< 0.001
Serum uric acid (umol/L)	362.1 ± 79.5	309.9 ± 81.1	< 0.001
Serum glucose (mmol/L)	6.1 ± 2.1	5.8 ± 1.8	< 0.001
Serum phosphorus (mmol/L)	1.15 ± 0.17	1.25 ± 0.16	< 0.001
Serum calcium (mmol/L)	2.35 ± 0.09	2.37 ± 0.10	< 0.001
*Serum 25(OH)D (%)*			< 0.001
≤ 49.9 nmol/L	6.4	5.3	
50.0–74.9 nmol/L	17.3	9.7	
≥ 75.0 nmol/L	21.0	28.6	
Not recorded	55.3	56.3	
Waist circumference (cm)	105.2 ± 13.0	97.3 ± 13.5	< 0.001
Femoral neck bone mineral density (mg/cm^2^)	794.5 ± 134.9	689.0 ± 127.2	< 0.001

Mean ± SD for continuous variables: *P* value was calculated by weighted linear regression model

% for categorical variables: *P* value was calculated by weighted chi-square test

The association between WC and femoral neck BMD for the four linear regression models is shown in Table 2. In models 1 and 2, WC was positively associated with femoral neck BMD. However, after adjusting for BMI, this association became negative (model 3: β = −0.7, 95% CI: −1.3, −0.2; model 4, β = −0.5, 95% CI: −1.1, −0.0). On subgroup analysis stratified by sex and race, the negative association only existed for men (β = −1.5, 95% CI: −2.4, -0.6) and Mexican Americans (β = −1.3, 95%CI: −2.6, −0.0).

**Table 2:** Association between waist circumference (cm) and femoral neck bone mineral density (mg/cm^2^) among old adults

	Model 1 (ß, 95% CI)	Model 2 (ß, 95% CI)	Model 3 (ß, 95% CI)	Model 4 (ß, 95% CI)
Waist circumference	3.6 (3.4, 3.9)***	2.6 (2.4, 2.8)***	−0.7 (−1.3, −0.2)**	−0.5 (−1.1, −0.0)*
*Waist circumference (Quartile)*				
Q1 (≤ 91.4)	Reference	Reference	Reference	Reference
Q2 (91.5–100.0)	67.5 (57.7, 77.3)	50.2 (41.2, 59.1)	18.2 (8.5, 27.8)	20.2 (10.7, 29.8)
Q3 (100.1–108.9)	97.1 (87.4, 106.9)	70.5 (61.4, 79.6)	14.4 (3.2, 25.7)	15.5 (4.2, 26.7)
Q4 (≥ 109.0)	132.1 (122.7, 141.6)	93.1 (84.0, 102.2)	−4.6 (−19.6, 10.4)	0.3 (−14.8, 15.3)
P for trend	< 0.001	< 0.001	0.685	0.929
*Stratified by sex*				
Men	2.6 (2.3, 3.0)***	2.7 (2.4, 3.1)***	−1.7 (−2.6, −0.9)***	−1.5 (−2.4, −0.6)***
Women	2.9 (2.6, 3.2)***	2.5 (2.2, 2.8)***	−0.4 (−1.0, 0.3)	0.0 (−0.7, 0.7)
*Stratified by race*				
Non-Hispanic White	3.7 (3.3, 4.0)	2.4 (2.1, 2.8)	−0.7 (−1.5, 0.1)	−0.4 (−1.2, 0.3)
Non-Hispanic Black	3.7 (3.1, 4.3)	3.4 (2.9, 4.0)	−0.3 (−1.5, 0.9)	−0.2 (−1.5, 1.0)
Mexican American	3.0 (2.3, 3.7)	1.9 (1.2, 2.5)	−1.3 (−2.7, 0.1)	−1.3 (−2.6, −0.0)*
Other race	4.4 (3.7, 5.0)	3.7 (3.1, 4.3)	−1.1 (−2.5, 0.2)	−0.8 (−2.2, 0.6)

Model 1: no covariates were adjusted

Model 2: age, sex, and race were adjusted

Model 3: model 2 plus body mass index were adjusted

Model 4: model 3 plus education level, income-poverty ratio, moderate recreational activities, alcohol use, smoking cigarettes, blood urea nitrogen, total protein, serum uric acid, serum glucose, serum phosphorus, serum calcium, and serum 25(OH)D were adjusted

In the subgroup analysis stratified by sex or race, the model is not adjusted for the stratification variable itself

*P < 0.05, **P < 0.01, ***P < 0.001

**Fig. 1:**
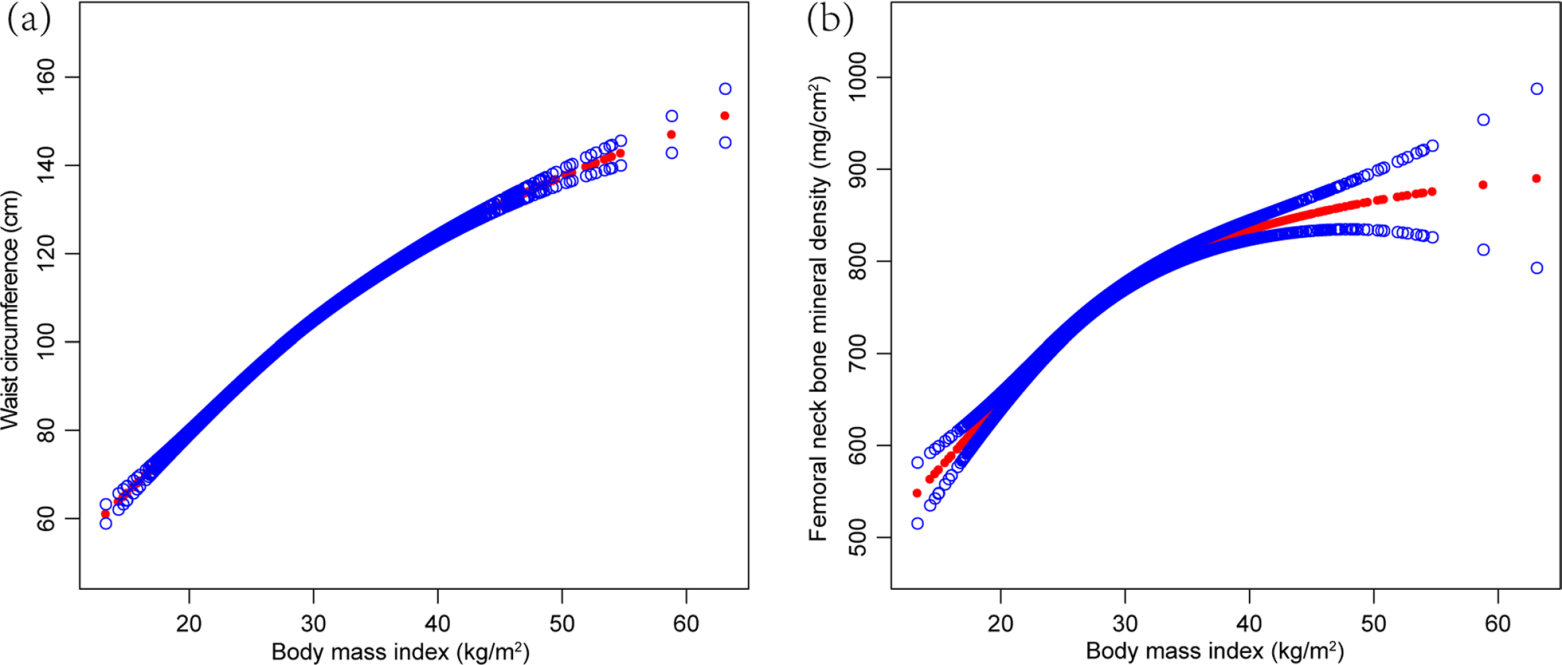
Association between body mass index and waist circumference and femoral neck bone mineral density. **a** Association between body mass index and waist circumference. **b** Association between body mass index and femoral neck bone mineral density

We evaluated the individual association between BMI and WC and femoral neck BMD, respectively, to clarify the mediating role of BMI on the association between WC and femoral neck BMD (Fig. 1), as well as performing a subgroup analysis stratified by BMI. In these analyses, the negative association between WC and femoral neck BMD existed in men for the BMI groups of 25–29.9 kg/m^2^ and ≥ 30 kg/m^2^ groups and in women for the BMI group ≥ 30 kg/m^2^ (Table 3).

**Table 3:** Association between waist circumference (cm) and femoral neck bone mineral density (mg/cm^2^) among older adults, stratified by body mass index

	Model 1 (ß, 95% CI)	Model 2 (ß, 95% CI)	Model 3 (ß, 95% CI)	Model 4 (ß, 95% CI)
*Men*				
BMI (< 25 kg/m^2^) (n = 762)	2.0 (0.8, 3.2)***	3.1 (1.9, 4.2)***	−1.5 (−3.2, 0.2)	−1.6 (−3.4, 0.1)
BMI (25–29.9 kg/m^2^) (n = 1,277)	−1.3 (−2.4, −0.2)*	−0.0 (−1.2, 1.1)	−2.2 (−3.5, −0.9)**	−2.0 (−3.4, −0.7)**
BMI (≥ 30 kg/m^2^) (n = 932)	0.7 (−0.2, 1.7)	0.9 (−0.1, 1.8)	−2.5 (−4.1, −0.9)**	−1.7 (−3.3, −0.0)*
*Women*				
BMI (< 25 kg/m^2^) (n = 788)	2.4 (1.5, 3.4)***	2.4 (1.5, 3.3)***	0.6 (−0.7, 1.8)	0.7 (−0.6, 2.0)
BMI (25–29.9 kg/m^2^) (n = 1,012)	0.4 (−0.6, 1.5)	0.7 (−0.3, 1.7)	0.1 (−1.1, 1.2)	0.4 (−0.8, 1.5)
BMI (≥ 30 kg/m^2^) (n = 1,030)	−0.1 (−0.9, 0.7)	−0.4 (−1.2, 0.4)	−2.4 (−3.6, −1.3)***	−2.1 (−3.2, −0.9)***

Model 1: no covariates were adjusted

Model 2: age and race were adjusted

Model 3: model 2 plus body mass index were adjusted

Model 4: model 3 plus education level, income-poverty ratio, moderate recreational activities, alcohol use, smoking cigarettes, blood urea nitrogen, total protein, serum uric acid, serum glucose, serum phosphorus, serum calcium, and serum 25(OH)D were adjusted

BMI, body mass index

*P < 0.05, **P < 0.01, ***P < 0.001

**Fig. 2:**
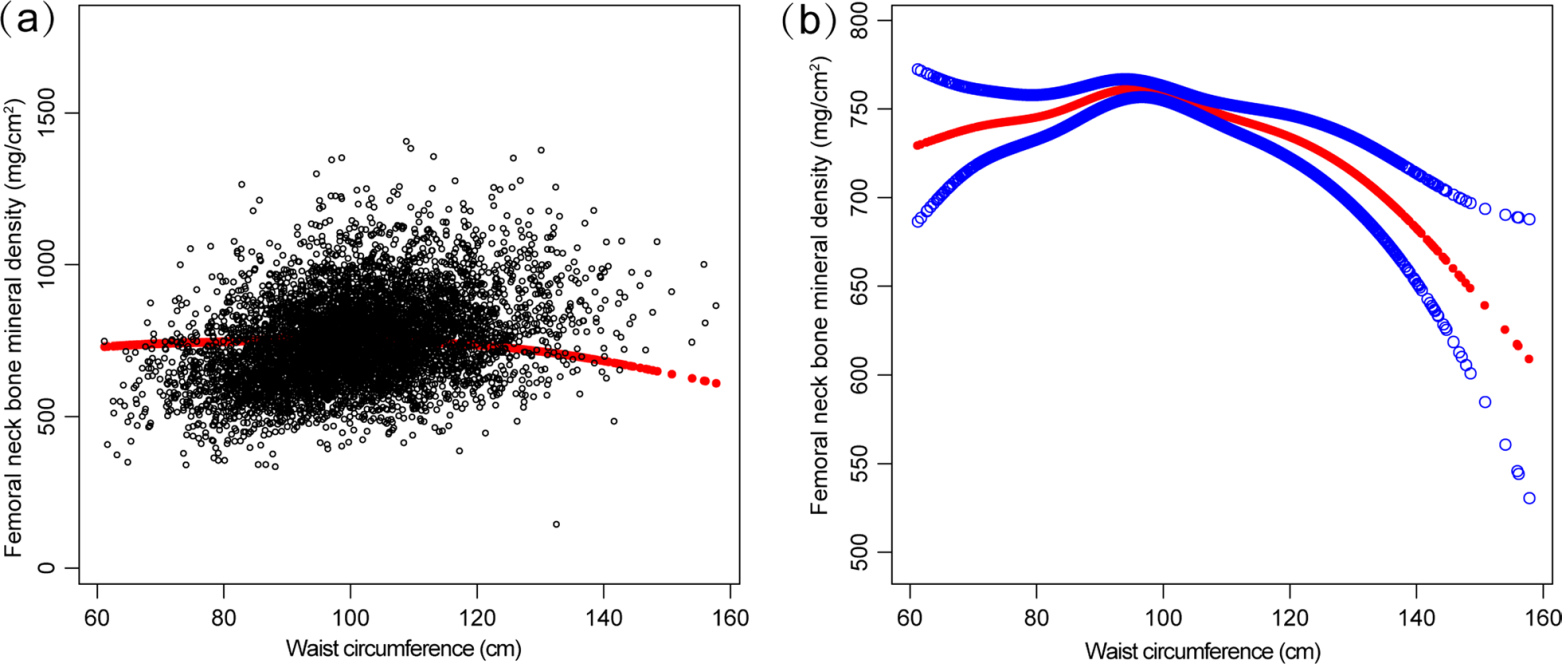
Association between waist circumference and femoral neck bone mineral density. **a** Each black point represents a sample. **b** Solid rad line represents the smooth curve fit between variables. Blue bands represent the 95% of confidence interval from the fit. Age, sex, race, body mass index, education level, income-poverty ratio, moderate recreational activities, smoking, alcohol use, smoking cigarettes, blood urea nitrogen, total protein, serum uric acid, serum glucose, serum phosphorus, serum calcium, and serum 25(OH)D were adjusted

**Fig. 3:**
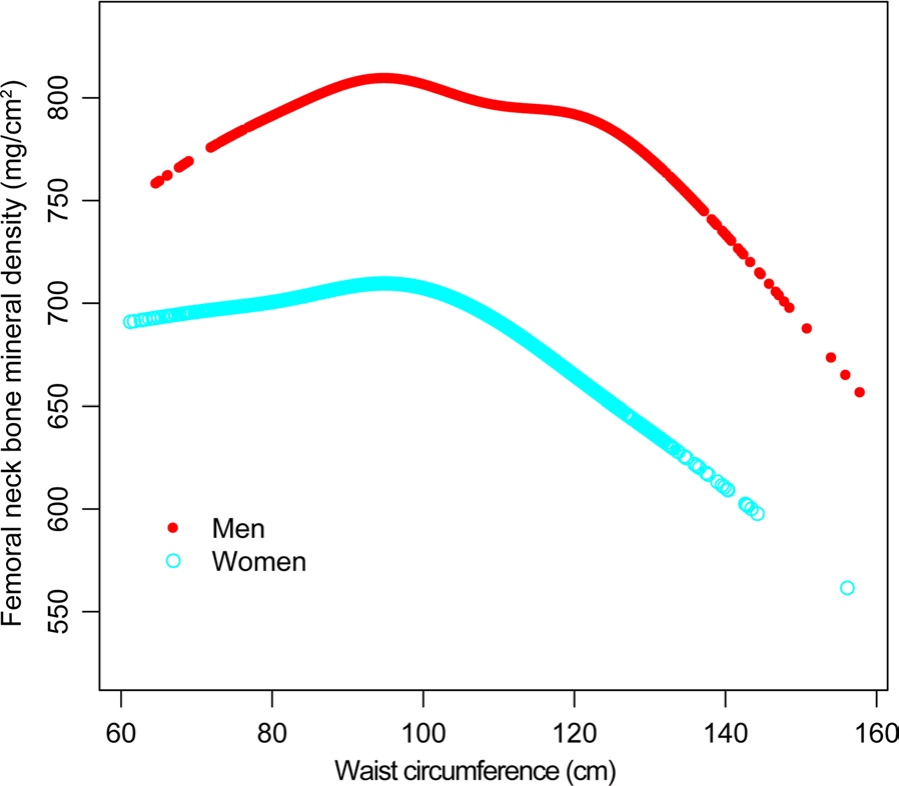
Association between waist circumference and femoral neck bone mineral density, stratified by sex. Age, race, body mass index, education level, income-poverty ratio, moderate recreational activities, smoking, alcohol use, smoking cigarettes, blood urea nitrogen, total protein, serum uric acid, serum glucose, serum phosphorus, serum calcium, and serum 25(OH)D were adjusted

Curve fitting for nonlinearity revealed an inverted U-shaped curve relationship between WC and femoral neck BMD in both men and women (Figs. 2 and 3). The inflection point was identified at 95 cm for both men and women (Table 4).

**Table 4:** Threshold effect analysis of waist circumference (cm) on femoral neck bone mineral density (mg/cm^2^) in older adults using two-piecewise linear regression model

Femoral neck bone mineral density	Adjusted ß (95% CI)
*Men*	
Fitting by standard linear model	−1.5 (−2.4, −0.6) < 0.001
Fitting by two-piecewise linear model	
Inflection point	95
Waist circumference < 95 (cm)	1.3 (−0.1, 2.8) 0.068
Waist circumference > 95 (cm)	−2.5 (−3.4, −1.5) < 0.001
Log likelihood ratio	< 0.001
*Women*	
Fitting by standard linear model	0.0 (−0.7, 0.7) 0.987
Fitting by two-piecewise linear model	
Inflection point	95
Waist circumference < 95 (cm)	1.6 (0.7, 2.5) < 0.001
Waist circumference > 95 (cm)	−1.6 (−2.5, −0.7) < 0.001
Log likelihood ratio	< 0.001

Age, race, body mass index, education level, income-poverty ratio, moderate recreational activities, alcohol use, smoking cigarettes, blood urea nitrogen, total protein, serum uric acid, serum glucose, serum phosphorus, serum calcium, and serum 25(OH)D were adjusted

## Discussion

In this study, we retrospectively investigated the association between WC, as a clinical parameter of abdominal obesity, and femoral neck BMD among older adults. We identified a U-shaped association between these two variables, with a point of inflection at a WC of 95 cm in both men and women. A previous study reported that general obesity was associated with a significantly higher BMD among healthy-weight individuals, suggestive of a protective effect of obesity, defined by the BMI, for osteoporosis [14]. A recent meta-analysis of 121 studies also reported higher lumbar spine, total hip, femoral neck, and radius BMD among men and premenopausal and postmenopausal women compared to their non-obesity counterparts [15]. However, obesity is no longer defined by BMI alone, with abdominal adiposity, measured by the WC, having become widely accepted as a better predictor of several adverse health outcomes [16, 17]. However, data regarding the association between abdominal obesity and BMD are scarce and inconsistent.

A rural community study conducted in Taiwan reported a negative association between abdominal obesity and osteoporosis in all three logistic regression models [18]. In a cross-sectional study conducted in Turkey, the authors found that the association between WC and BMD differed by sites of BMD measurement, with a positive association at the hip but a negative association for non-weight-bearing sites [19]. By comparison, results of a Korean community-based study of individuals aged ≥ 50 years revealed that WC was independently and inversely associated with femoral neck and lumbar spine BMD, after adjusting for other body composition parameters, with this association being stronger in men than women [20]. This result was consistent with a previous Korean community-dwelling cohort study which reported a negative association between WC and bone mineral content after adjusting for weight [21]. In a cross-sectional study of 4,663 Chinese men of normal weight (BMI, 18.5–22.9 kg/m^2^), WC was identified as a negative predictor of calcaneal BMD [22]. The results of a survey of a nationally representative sample of elderly individuals in Israel showed a positive association between abdominal obesity and fragility fractures, independent of BMI, suggesting that WC may be a useful and easily measured anthropometric indicator for assessing the risk of osteoporotic fractures [23]. Our data showed a positive association between WC and femoral neck BMD in the non-BMI adjusted models and a negative association after adjusting for BMI, particularly in men. These different conclusions may be attributed to the study population, study design, BMD examination methods and sites of evaluation, and the control of confounding variables, especially BMI.

The exact mechanism for the deleterious effects of obesity on bone health remains unclear. Several mechanisms have been proposed: increased metabolism and accelerated senescence in stromal stem cells [24]; increased inflammation associated with obesity [25]; replacement of osteoblasts by fat cells in bone marrow [26]; and mutations in the fat mass and obesity-associated gene leading to bone fragility [27]. Further studies are needed to clarify the underlying mechanisms linking obesity and bone health. Considering the negative impact of abdominal obesity on bone health and other health conditions, lifestyle interventions and well-designed clinical trials are urgently needed for the prevention and management of abdominal obesity among older individuals.

The NHANES survey provided data from a nationally representative sample and this large sample size was sufficient to provide good statistical power. Nevertheless, several limitations of our study should be noted. First, definitive causal inferences could not be determined due to the inherent nature of a cross-sectional study. Second, lumbar spine BMD was not included in the analysis because the target population for lumbar spine BMD was aged 8–59 years. Third, participants with a history of cancer were excluded; therefore, the findings of our study do not apply to this clinical population. Fourth, the bias caused by residual confounding factors remains.

In summary, we identified abdominal obesity as a negative predictor of bone health among older adults, independent of BMI, with an inverted U-shaped association, and a point of inflection at a WC of 95 cm. Our findings indicate the need for effective weight-management strategies to lower the risk of age-related obesity and improve bone health.
